# Differential expression of functional chemokine receptors on human blood and lung group 2 innate lymphoid cells

**DOI:** 10.1016/j.jaci.2018.08.030

**Published:** 2019-01

**Authors:** Cathryn A. Weston, Batika M.J. Rana, David J. Cousins

**Affiliations:** a Department of Infection, Immunity and Inflammation, NIHR Leicester Biomedical Research Centre - Respiratory, Leicester Institute for Lung Health, University of Leicester, Leicestershire, United Kingdom; b MRC & Asthma UK Centre in Allergic Mechanisms of Asthma, King's College London, London, United Kingdom; c MRC Laboratory of Molecular Biology, Cambridge, United Kingdom

To the Editor:

Innate lymphoid cells (ILCs), primarily found at mucosal barriers, provide immediate protection against the establishment and spread of infection. ILCs have been divided into 3 subsets analogous to T_H_ cells^1^: ILC1, ILC2, and ILC3. ILC2s are similar to T_H_2 cells and express IL-4, IL-5, and IL-13 and were initially identified as a non–T-, non–B-cell source of type 2 cytokines.^2^ They are found in the blood, gut, skin, and lung where they contribute to host defence. Upon activation, ILCs rapidly produce a large quantity of cytokines and other mediators, which attract and activate other inflammatory cells. In various models of airway disease, ILC2 numbers have been shown to increase with allergen challenge, leading to a significant increase in type 2 inflammatory cytokines.^3^ Recent studies have demonstrated the existence of a complex interplay between lung epithelial cells and ILC2s that is required for asthma persistence in a mouse model. Furthermore, human studies have suggested that ILC2s provide the key link between viral infection and airway inflammation leading to asthma exacerbations.^4^

ILC2s are produced from precursor cells in the bone marrow and ILC precursors have been identified in human blood; however, there is debate about how ILCs populate the adult tissue, with some studies indicating that they are predominantly tissue-resident cells.^5^ An important question in ILC biology is to understand the mechanisms by which both progenitor and mature cells are recruited to the peripheral tissues such as the lung.

A significant class of cell surface receptors known to be important for immune cell migration is the chemokine receptors.^6^ Importantly, upregulation of key chemokines has been observed in the bronchial biopsies of patients with asthma following allergen challenge. Because of this there has been much interest in the possibility of developing antagonists, which inhibit receptor activation, to prevent unwanted cells from being recruited to sites of inflammation such as the lung during asthma exacerbation.^7^ Using flow cytometry we assessed the expression of chemokine receptors on T cells and ILC2s in both human blood and lung samples. See this article's Methods section in the Online Repository at www.jacionline.org. The mean data are represented in Fig 1 (11 blood donors and 5 lung samples). The pattern of receptor expression broadly agreed with previous reports for CD3^+^ T cells. A smaller subset of receptors tested was readily detectable on ILC2s. These data were reflected at the RNA level as confirmed using real-time PCR probes to detect the different chemokine receptors in cDNA synthesized from RNA isolated from fresh ILC2 and T cells (see Fig E4 in this article's Online Repository at www.jacionline.org).

**Fig 1:**
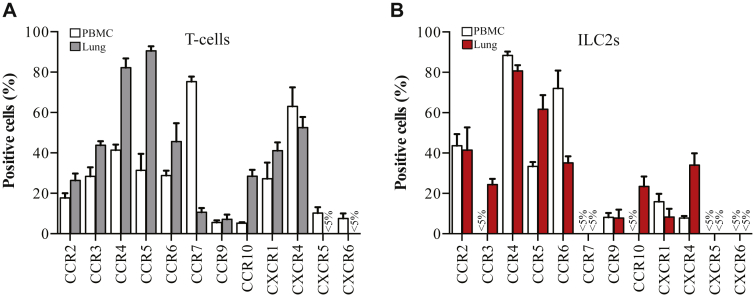
ILC2s and T cells isolated from human lung display different chemokine receptors to those derived from blood. **A,** Percentage of CD3^+^ T cells from blood (white) and lung tissue (gray) expressing the indicated chemokine receptors. **B,** Percentage of ILC2s from blood (white) and lung (red) expressing the indicated chemokine receptors. Data are mean ± SEM of 11 (PBMCs) and 5 (lung) independent donors.

Although highly detectable on blood-derived T cells, CCR7 was not detected on ILC2s isolated from the blood or lung. CCR7 expression has previously been described on lymphocyte precursors within the bone marrow but is maintained only on mature ILC1 and ILC3s, in particular subsets of ILCs found in the spleen and lymph nodes.^8^ Our data suggest that CCR7 does not play a role in the trafficking of mature ILC2s in the blood or lung tissues. CXCR5 and CXCR6 were expressed only on a small subset of blood T cells and even fewer (<5%) lung T cells. Furthermore, CXCR5 and CXCR6 were detected on less than 5% of ILC2s isolated from either the blood or lung. These results are consistent with the notion that CXCR5 is largely involved in B-cell homing and that CXCR6 is important for the retention of T cells in the liver, and for the emigration of ILC3s from the bone marrow to the small intestine.^8^ CCR9, thought to direct cells to the small intestine, was detectable on only around 10% of ILC2s in lung tissue.

Several receptors were significantly (*P* < .05) upregulated in lung tissue–derived ILC2s compared with the cells isolated from the blood including CCR3 and CXCR4 (Fig 1). CCR3 in combination with CCR4 has been shown in multiple T-cell studies^7^ to regulate recruitment to the lung. Furthermore, the potent inflammatory ligand (CXCL12) acting via CXCR4 appears to coordinate with CCL11 activation of CCR3 and CCL22 stimulation of CCR4 to recruit lymphocytes to the lung and generate an inflammatory reaction.^7^ Our data indicate that a similar mechanism could be used to activate or recruit ILC2s to the airways, thereby driving inflammation.

The receptors displayed on the highest proportion of ILC2s isolated from both blood and lung tissues were CCR2, CCR4, CCR5, and CCR6. We therefore wished to determine whether these receptors could be used to activate blood ILC2s via various chemokine ligands. Because ILC2s are found only as a low percentage of human PBMCs, traditional chemotaxis assays would have been difficult to reliably perform. We therefore used an actin polymerization assay as a marker of receptor activation. The ligands chosen for this assay (see Table E2 in this article's Online Repository at www.jacionline.org) have all been detected in the lung^7^ and were based on their receptor specificity. The stimulation time was optimized to give the largest signal window (see Fig E5 in this article's Online Repository at www.jacionline.org). Both ILC2s and T cells displayed ligand-dependent increases in their F-actin content following 10-second stimulation with the chemokines (Fig 2). Because of the short duration of the assay, it is unlikely that these effects are through indirect activation of other immune cells. The potency for each ligand was similar between the T cells and ILC2s; however, the maximal responses were significantly (*P* < .05) different for all but CCL2 stimulation of CCR2, perhaps indicating differences in the number of the receptors expressed on each cell type. Comparing the response of ILC2s to each ligand (Fig 2, *E*; see Table E2 in this article's Online Repository at www.jacionline.org) reveals that the strongest response is seen via CCR4, with CCR5 and CCR6 showing similar lower levels of activation and CCR2 an intermediary response. These values may reflect differences in receptor number on the ILC2 cell surface and may correspond to their roles in cell activity and tissue recruitment because it is thought that a higher receptor number is required to achieve cell chemotaxis. In T cells CCR5 does not direct tissue-specific recruitment but is required in combination with different panels of receptors to enable migration.^7^ Therefore, it may be that initial activation of ILC2s by the more highly expressed receptors occurs before chemotaxis is enhanced through the binding of CCR5-specific ligands.

**Fig 2:**
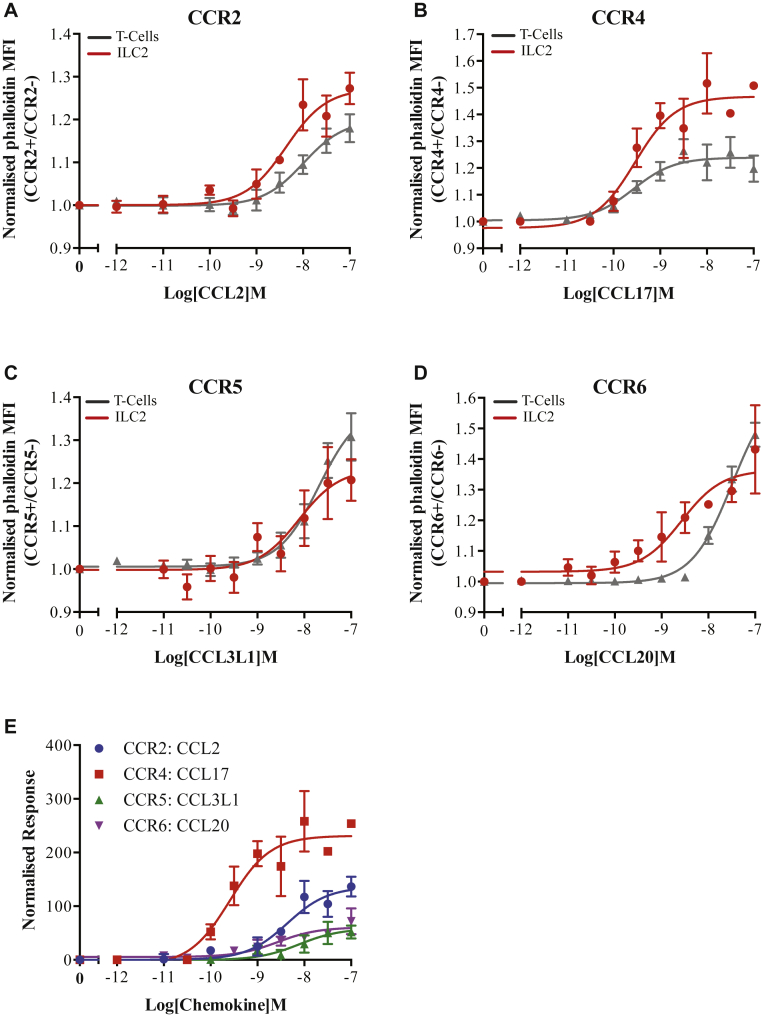
ILC2s respond to various chemokine receptor ligands in a dose-dependent manner. Activation of **(A)** CCR2, **(B)** CCR4, **(C)** CCR5, and **(D)** CCR6 receptors on ILC2s and T cells was determined following 10-second stimulation with a range of concentrations of the indicated ligands. **E,** Normalized ILC2 response for each receptor/ligand combination. All data are mean ± SEM from 5 independent experiments. *MFI*, Mean fluorescence intensity.

Given that a higher proportions of “activated” IL-5^+^, IL-13^+^ ILC2s have been shown to correlate with asthma severity^9^ and that mouse models of asthma demonstrate that an increase in ILC2 number is sufficient for airway hyperresponsiveness, targeting the activation and recruitment of these cells is an attractive treatment strategy. Our data provide new insight into the potential mechanisms by which ILC2s may be recruited or activated in the human blood and lung, and may therefore allow rational selection of future therapeutics targeting ILC2s in airway disease.

## Figures and Tables

**Fig E1:**
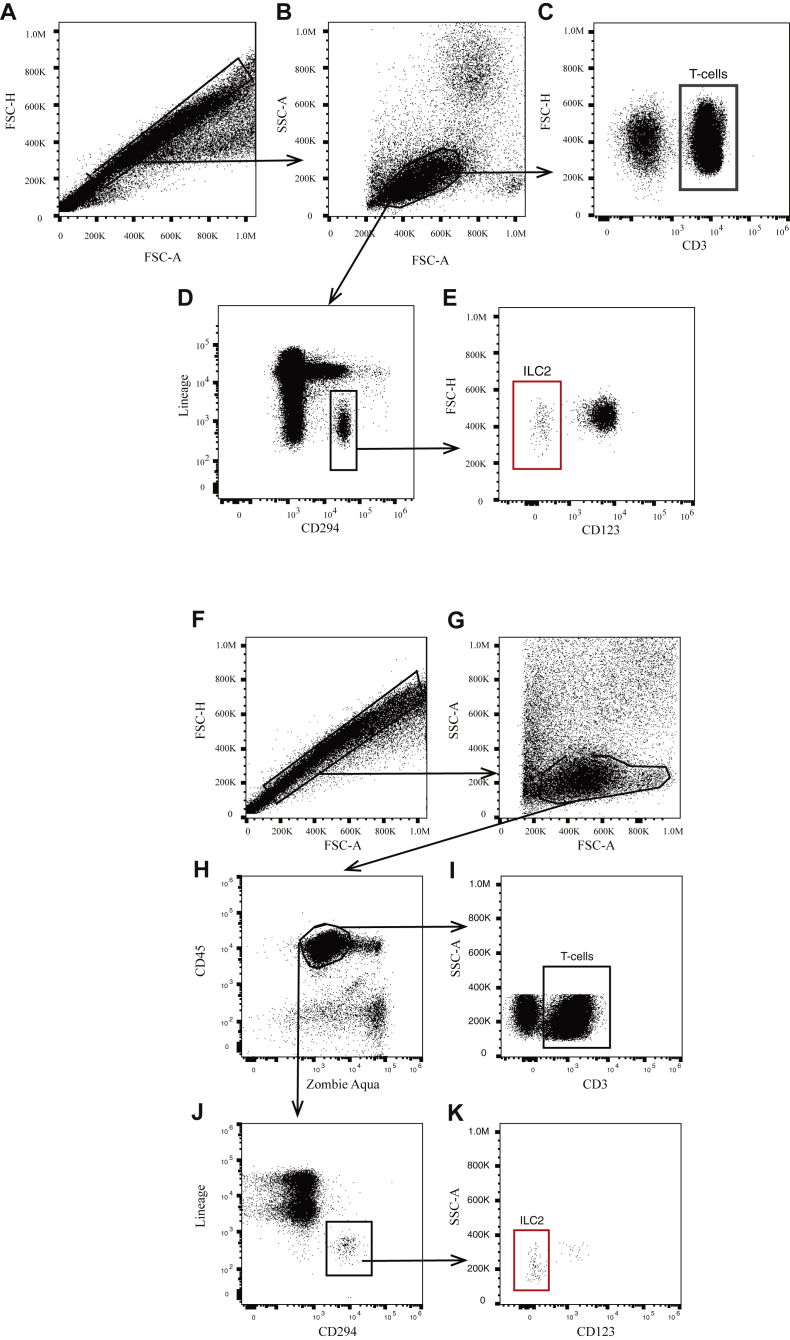
Gating strategy to identify ILC2s and T cells from human peripheral blood **(A-E)** and lung **(F-K)**. **A,** Total PBMCs displayed with singlet gate overlaid. **B,** Lymphocytes (gated cells) selected from single-cell population based on light scattering properties. **C,** T cells identified as the CD3^+^ population of single-cell lymphocytes. **D,** Lineage^−^, CD294^+^ cells (boxed region) containing the CD123^−^ ILC2s were also selected from the single-cell lymphocyte population **(E)**. **F,** Total cells displayed with singlet gate overlaid. **G,** Lymphocytes (gated cells) selected from single-cell population based on light scattering properties. **H,** Live (zombie aqua^−^), CD45^+^ cells shown in gated region were selected and T cells were identified as the CD3^+^ population **(I)**. **J,** Lineage^−^, CD294^+^ cells (boxed region) containing the CD123^−^ ILC2s were also selected from the live, CD45^+^, single-cell lymphocyte population **(K)**. *FSC-A*, Forward scatter-area; *FSC-H*, forward scatter-height; *SSC-A*, side scatter-area.

**Fig E2:**
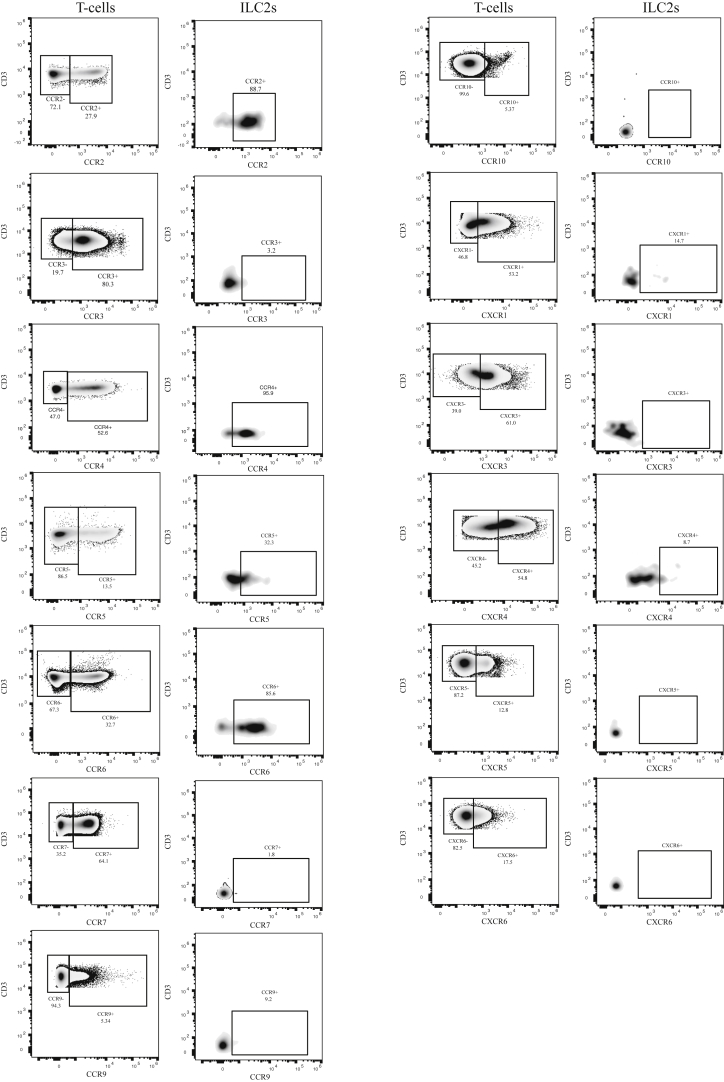
Chemokine receptor expression on T cells and ILC2s in human blood. Representative flow cytometry data showing the expression of the indicated chemokine receptors on CD3^+^ T cells (left) and ILC2s (right). Receptor^+^ gate determined using bimodal distribution observed on T-cell populations; numbers within gates indicate percentage cells.

**Fig E3:**
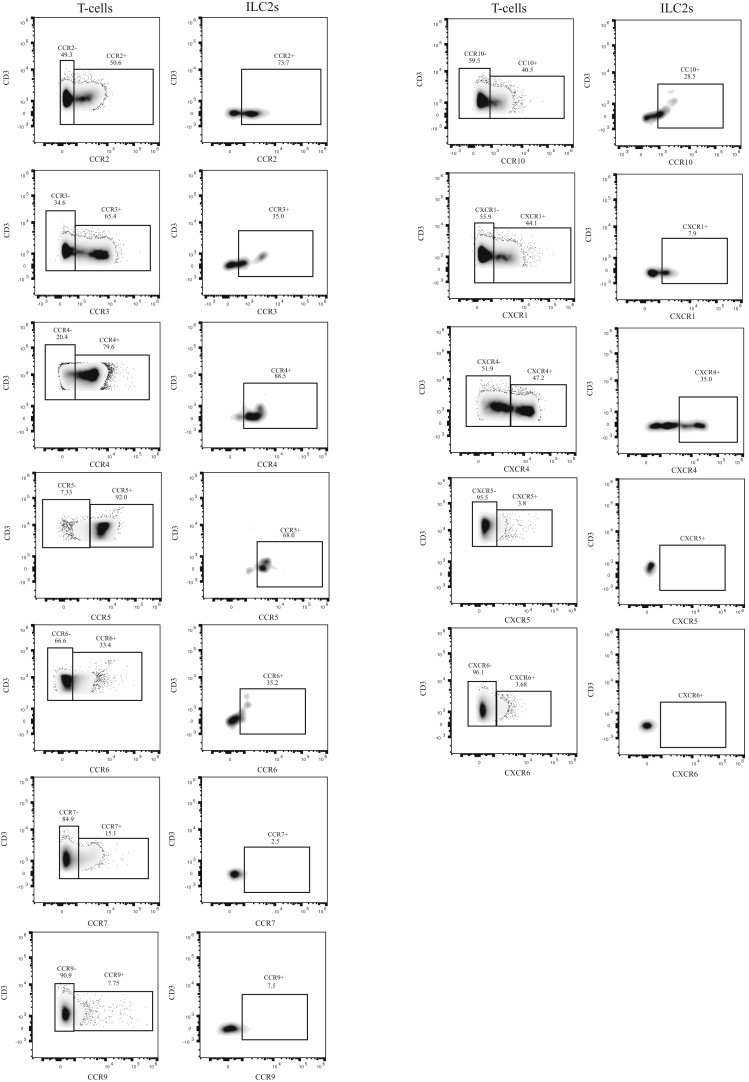
Expression of chemokine receptors on ILC2s and T cells isolated from lung tissue. Representative flow cytometry data showing the expression of the indicated chemokine receptors on CD3^+^ T cells (left) and ILC2s (right). Receptor^+^ gate determined using bimodal distribution observed on T-cell populations; numbers within gates indicate percentage cells.

**Fig E4:**
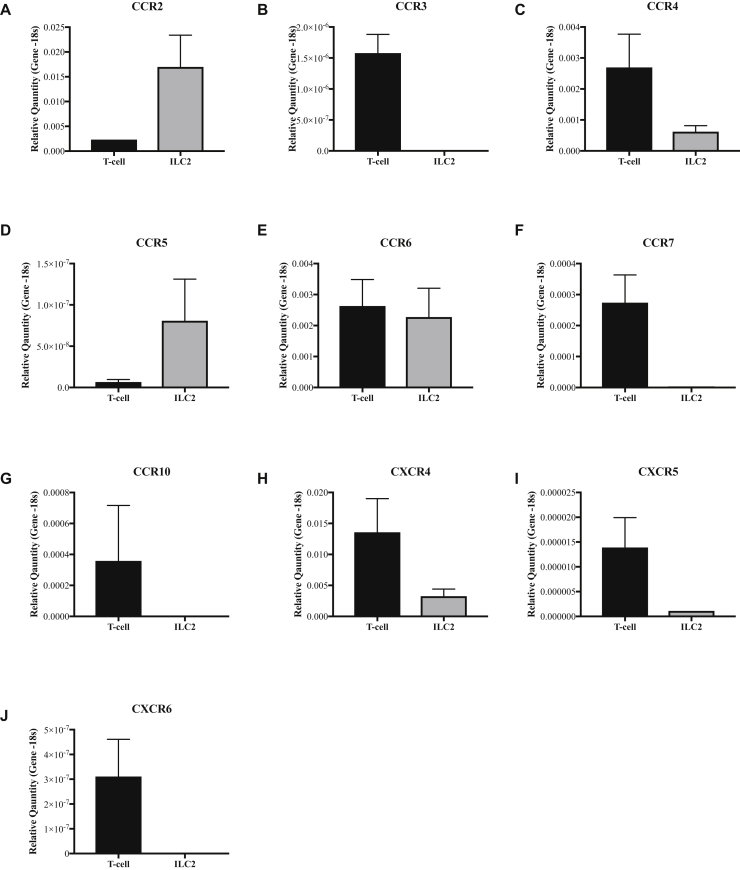
**A-J,** Quantification of chemokine receptor RNA expression in T cells and ILC2s. Relative quantities of the indicated genes were calculated relative to 18S mRNA. Plots show mean values (±SEM) for T cells (black boxes) and ILC2s (gray boxes) sorted from PBMCS obtained from 5 independent donors.

**Fig E5:**
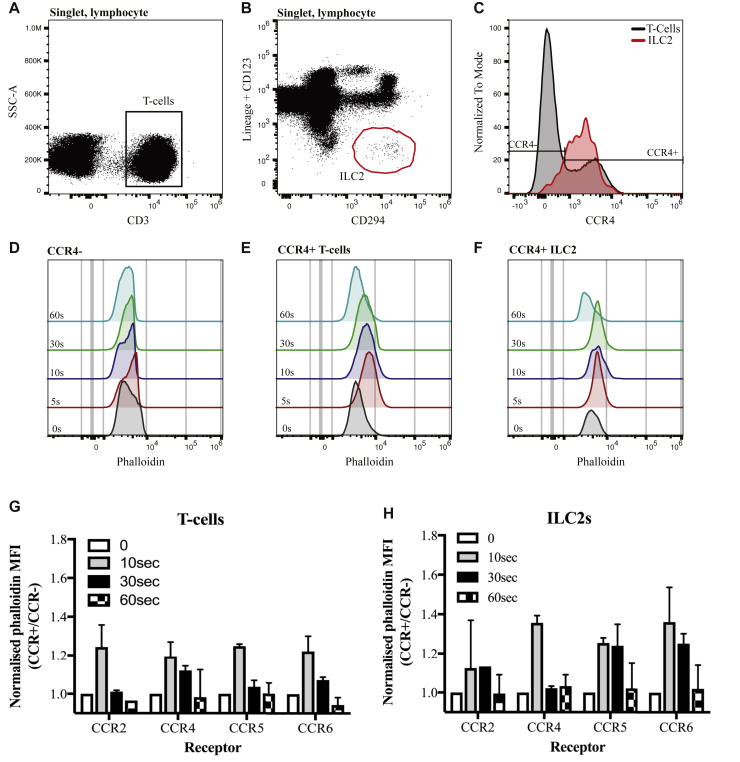
Chemokines can be used to stimulate human blood cells in a time-dependent manner. **(A)** CD3^+^ T cells and **(B)** Lineage^−^, CD123^−^, CD294^+^ ILC2s were identified from within a single-cell, lymphocyte gate. **C,** CCR4 expression on T cells (gray) and ILC2s (red) with gates used to select positive and negative populations overlaid. Phalloidin staining was analyzed for **(D)** CCR4^−^ T cells, **(E)** CCR4^+^ T cells, and **(F)** CCR4^+^ ILC2s at the indicated time points. Receptor response was determined for **(G)** chemokine receptor–positive T cells and **(H)** chemokine receptor–positive ILC2s at each time point by normalizing the phalloidin MFI to that observed on CCR4^−^ cells. Data shown are mean ± SEM of 5 independent donors. *MFI*, Mean fluorescence intensity; *SSC-A*, side scatter-area.

**Table E1:** Taqman probes used in this study

Gene name	Probe ID	Gene name	Probe ID
CCR2	Hs00356601_m1	CCR7	Hs01013469_m1
CCR3	Hs00266213_s1	CCR10	Hs00706455_s1
CCR4	Hs00356611_s1	CXCR4	Hs00237052_m1
CCR5	Hs00152917_m1	CXCR5	Hs00540548_s1
CCR6	Hs01890706_s1	CXCR6	Hs01890898_s1

**Table E2:** Potency (pEC50) and maximal (*E*_max_) responses observed in ILC2s and T cells following 10-s stimulation with the indicated receptor:ligand combinations

	CCR2:CCL2		CCR4:CCL17		CCR5:CCL3L1		CCR6:CCL20	
	ILC2	T cell	ILC2	T cell	ILC2	T cell	ILC2	T cell
**pEC50**	8.4 ± 0.2	8.0 ± 0.2	9.6 ± 0.2	9.6 ± 0.2	8.1 ± 0.4	7.7 ± 0.2	8.6 ± 0.3*	7.5 ± 0.1
*E*_max_	1.3 ± 0.03	1.2 ± 0.02	1.5 ± 0.04***	1.2 ± 0.02	1.2 ± 0.06**	1.4 ± 0.04	1.4 ± 0.04**	1.6 ± 0.05

Data are mean ± SEM of experiments from at least 5 independent donors. Statistical significance was determined where the ILC2 data set was compared to the T cell data for each receptor:ligand combination (*P < .05, **P < .001, ***P < .0001).

## Methods

### PBMC isolation

PBMCs were obtained from whole-blood samples taken from healthy, nonatopic subjects with no evidence of parasitosis recruited at University Hospitals of Leicester NHS Trust, Leicester, UK, who had provided informed written consent, under ethics (Research ethics committee [REC] reference number 08/H0406/189) approved by the Leicestershire, Northamptonshire, and Rutland ethics committee. The heparinized blood was mixed with an equal volume of PBS and centrifuged (800*g*, 20 minutes, 22^o^C) over Lymphoprep (Stem Cell Technologies, Cambridge, UK). Isolated PBMCs were washed in PBS containing 2% FBS (Thermofisher, Paisley, UK) before counting using a hemocytometer and trypan blue stain to enable resuspension at the concentration required for further experiments as described below.

### Isolation of cells from lung tissue

Lung cells were isolated from healthy lung resection material taken from patients who had provided informed written consent and were undergoing lung surgery. This material was ethically obtained at University Hospitals of Leicester NHS Trust, under the auspices of the Midlands lung tissue consortium (REC reference number 07/MRE08/42). Lung tissue bathed in Dulbecco modified Eagle medium containing 2% FBS was enzymatically digested for 75 minutes at 37^o^C using hyaluronidase (0.75 mg/mL, H3506, Sigma, Dorset, UK) and collagenase (0.75 mg/mL, C2674, Sigma) within 1 hour of resection. The digested tissue was filtered by sequentially passing through first a 100-μm gauze and then a 50-μm gauze, and washed twice with Dulbecco modified Eagle medium containing 2% FCS (centrifuging at 230*g* for 8 minutes, 4°C). Cell number was determined using hemocytometer and Kimura stain before washing in the absence of serum and resuspending in PBS at a concentration of 1 × 10^7^/100 μL for flow cytometric analysis as described below.

### Analysis of cell surface markers by flow cytometry

For each condition tested, 100 μL (1 × 10^7^ cells) of lung tissue cells was first stained for 20 minutes with Zombie Aqua fixable viability kit following the manufacturer's protocol (Biolegend, London, UK). Following centrifugation (200*g*, 10 minutes, 4^o^C), the stained cells were resuspended in 100 μL brilliant violet staining buffer (Beckton Dickinson, Oxford, UK). PBMCs were adjusted to a concentration 1 × 10^6^/100 μL before cell surface staining. Cells were first incubated with Human Trustain (Biolegend) at room temperature for 15 minutes to block Fc receptors. mAbs: fluorescein isothiocyanate–conjugated lineage cocktail (α-CD2, α-CD3, α-CD14, α-CD16, α-CD19, α-CD56, α-CD235a), EF450-conjugated α-CD3 (UCHT1) (both Affymetrix, Cheshire, UK), BV605-conjugated CD123 (6H6), AF647-conjugated α-CD294 (BM16), α-CCR2 (CD192; K036C2), α-CCR4 (CD194; L291H4), α-CCR5 (CD195; JF418F1), α-CCR6 (CD196; G034E3), α-CCR7 (CD197; G043H7), α-CCR9 (CD199; L053E8), α-CXCR1 (CD181; 8F1/CXCR1), α-CXCR3 (CD183; G025H7), α-CXCR4 (CD184; 12G5), α-CXCR5 (CD185; J252D4), α-CXCR6 (CD186; K041E5) all phycoerythrin (PE)/Cy7-conjugated, PE-conjugated α-CCR3 (CD193; 5E8), and α-CCR10 (6588-5) (all Biolegend) were then added for a further 20-minute incubation at room temperature. For lung tissue–derived cells, BV785-conjugated α-CD45 (HI30, Biolegend) was also added. Cells were then fixed and any red blood cells lysed through the addition of a 1-step fix and lyse solution for 20 minutes at room temperature (Affymetrix) before centrifugation at 300*g* for 10 minutes, 4^o^C, and resuspension in PBS supplemented with 2% FBS for acquisition on a 4-laser Attune NxT flow cytometer using NxT software v2.2 (Life Technologies, Paisley, UK). Compensation settings were determined using unstained cells and UltraComp eBeads compensation beads (Affymetrix). Following the exclusion of doublets (Fig E1, *A*), lymphocytes were selected on the basis of their light scatter properties (Fig E1, *B*). From the selected lymphocyte population, both CD3^+^ T cells (Fig E1, *C*) and lineage^−^, CD123^−^, CD294^+^ ILC2s were identified (Fig E1, *D* and *E*). CD45 was added as an additional positive marker for identifying T-cell and ILC2 populations in lung tissue (Fig E1, *G*). Because of the observed bimodal distribution of expression, the CD3^+^ and chemokine receptor–positive population was used to determine the proportion of ILC2s also displaying each chemokine receptor (Figs E2 and E3, histograms).

### Real-time PCR

T cells and ILC2s were sorted from PBMCs prepared as for flow cytometric analysis using a FACS ARIAIII (Beckton Dickinson) directly into TRIZOL LS (ThermoFisher, Paisley, UK). RNA was isolated from the purified cell populations using the miRNeasy mini kit (Qiagen, Manchester, UK), followed by DNAse digestion (Thermofisher) and RNeasy minElute cleanup (Qiagen) according to the respective manufacturer's instructions. RNA integrity was assessed using a bioanalyzer (Agilent, Edinburgh, UK), according to the manufacturer's instructions. Because of low cell number, cDNA samples were synthesized and amplified using the Ovation V2 kit (NuGEN, Leek, Netherlands) as per the manufacturer's instructions. Real-time RT-PCR was performed using TaqMan Master mix II (ThermoFisher) and TaqMan probesets (all FAM-MGB) listed in Table E1. The chemokine receptor signals were compared with an 18S VIC-MGB rRNA endogenous control (ThermoFisher). Cycling conditions were as follows: 50°C for 2 minutes, 95°C for 10 minutes, 95°C for 15 seconds, and then 60°C for 1 minute for 50 cycles on a QuantStudio 5 system (ThermoFisher).

### Measurement of cellular F-actin content following chemokine stimulation

Isolated PBMCs were adjusted to 1 × 10^7^cells/100 μL. The ability of various recombinant chemokines (CCL2, CCL17, CCL3L1, CCL20, all Biotechne, Abingdon, UK) to activate their cognate receptor was then tested through measurement of chemokine-induced increases in the filamentous (F)-actin content of both CD3^+^ T cells and ILC2s. The method has previously been demonstrated as a marker for T-cell activation.E1, E2 Briefly, 100 μL of cells per 10-point dose response curve was taken and Fc receptors were blocked and stained with fluorescein isothiocyanate–conjugated CD123 and lineage cocktail, AF647-conjugated CD294, EF450-conjugated CD3, and the required PE/cy7-conjugated chemokine receptor antibodies for 20 minutes at room temperature. Cells were washed in PBS containing 2% FBS and then resuspended in prewarmed (37^o^C) RPMI 1640 (ThermoFisher) containing 2% FBS before stimulation for 10 seconds with the appropriate chemokine. The reaction was terminated by the addition of an equal volume of fixation buffer from the intracellular fixation and permeabilization buffer set (Affymetrix) for 30 minutes at room temperature. Cells were washed (1200*g*, 5 minutes) 3 times with 1 × permeabilization buffer (from the intracellular fixation and permeabilization buffer set; Affymetrix) and stained with AF555-conjugated phalloidin (Life Technologies) (30 minutes at room temperature) before washing and resuspension in PBS containing 2% FBS. Data were acquired using an Attune NxT cytometer running NxT software v2.2. Chemokine-induced changes in F-actin content were quantified as an increase in the mean fluorescence intensity in the AF555 channel. To account for photobleaching and other nonspecific effects on AF555 fluorescence, the mean fluorescence intensity for chemokine receptor–positive cells was expressed as a fraction of that observed in the population not displaying the receptor for each sample.

### Data analysis

Analysis of flow cytometry data was performed using FlowJo v10 (Tree Star, Ashland, Ore). Concentration-response curves were fitted using the 3-parameter logistic equation in Graph Pad PRISM v6 (San Diego, Calif) to obtain EC_50_ values. Statistical analysis was performed using a 2-way ANOVA and Tukey multiple comparison test with a probability (*P*) of less than .05 being considered significant.
